# Effect of Interface Modification on Mechanoluminescence-Inorganic Perovskite Impact Sensors

**DOI:** 10.3390/s23010236

**Published:** 2022-12-26

**Authors:** Lucas Braga Carani, Vincent Obiozo Eze, Okenwa Okoli

**Affiliations:** High-Performance Materials Institute, FAMU-FSU College of Engineering, 2525 Pottsdamer Street, Tallahassee, FL 32310, USA

**Keywords:** structural health monitoring, composites, mechanoluminescence, inorganic perovskite, interface modification

## Abstract

It is becoming increasingly important to develop innovative self-powered, low-cost, and flexible sensors with the potential for structural health monitoring (SHM) applications. The mechanoluminescence (ML)-perovskite sensor is a potential candidate that combines the light-emitting principles of mechanoluminescence with the light-absorbing properties of perovskite materials. Continuous in-situ SHM with embedded sensors necessitates long-term stability. A highly stable cesium lead bromide photodetector with a carbon-based electrode and a zinc sulfide (ZnS): copper (Cu) ML layer was described in this article. The addition of a magnesium iodide (MgI_2_) interfacial modifier layer between the electron transport layer (ETL) and the Perovskite interface improved the sensor’s performance. Devices with the modified structure outperformed devices without the addition of MgI_2_ in terms of response time and impact-sensing applications.

## 1. Introduction

Structural health monitoring (SHM) aims to ensure the diagnostics of the host structure’s conditions, as well as its safety and integrity, on a continuous basis. The advancement of SHM technologies is inextricably linked to the overall safety of structures. Real-time SHM systems for advanced composite structures are in high demand [1,2,3]. To meet the demand, a flexible mechanoluminescent (ML)-perovskite impact sensor is proposed [4,5,6,7,8]. The device consists of a photodiode constructed on top of an ML layer. ML materials emit light in response to mechanical stimuli. The active absorber layer of a photodiode is in charge of light absorption and charge transport in order to generate electric signals. The electrical signals can then be analyzed to determine the condition of the structure. In practice, the ML layer would respond to load or impact events by emitting light into the photodetector device, which would then convert the light into electric signals that could be conditioned and interpreted for health monitoring [4,5,6]. Due to their exceptional optoelectronic properties, such as high charge carrier mobility, low exciton binding energy, tunable bandgap, and long carrier diffusion lengths, perovskite-based photodetectors appeared to be the most promising candidate for the active layer. Organic–inorganic hybrid perovskite solar cells have a power conversion efficiency (PCE) that exceeds 25.2%, outperforming most polycrystalline silicon solar cells [9,10,11,12,13].

Unfortunately, the organic–inorganic lead halide perovskites decompose due to chemical instability and susceptibility to moisture, heat, oxygen, and other environmental conditions. To address the aforementioned instability, an all-inorganic perovskite active layer is an alternative and promising candidate. CsPbX_3_ (X = Br, I) all-inorganic cesium-lead halide perovskites are gaining popularity due to their ability to increase a device’s inherent stability by replacing volatile organic cations with an inorganic cesium cation (Cs^+^) [14,15]. All inorganic CsPbBr_3_ materials are promising for optoelectronics due to their excellent light absorption, high charge carrier mobility, and long carrier diffusion length. Aside from its optoelectronic properties, the CsPbBr_3_ could withstand high temperatures of up to 580 °C, whereas the more common perovskite MAPbI_3_ began to lose performance at around 200 °C.

Furthermore, the CsPbBr_3_ perovskite solar cells barely deteriorated after three months in humid air [16,17,18]. High leakage current due to poor morphology (high pinhole density), significant non-radiative recombination at the perovskite/injection layers and within the perovskite layer itself, and charge-injection imbalance may all contribute to the low efficiency of some inorganic perovskite devices, limiting their use in SHM applications [15,19]. Several CsPbBr_3_ photodetectors have been reported, but their performance is still lacking.

In high-performance organic–inorganic perovskite solar cells, interfacial contact is critical. The interfacial contact is directly related to the chemical interaction between the perovskite and the electron transport layer (ETL). Interfacial contact is also important in the development of high-performance all-inorganic perovskites [14]. It is worth noting that exciton formation, dissociation, and recombination all have a direct relationship with the interface. Furthermore, the interface has a significant effect on device degradation. Guerrero and colleagues demonstrated that ionic movement in the perovskite layer causes interfacial metal contact degradation [20].

Several techniques have been used to reduce defect levels in perovskite films, including additive passivation, heterojunction engineering, and interface modification [10,18,20,21,22]. Yan developed a novel intermediate-phase engineering technique to increase the interfacial contact between all-inorganic perovskite and metal oxide by incorporating volatile organic salts into the inorganic perovskite precursor solution, resulting in a PCE of 17% [22]. By incorporating a trace amount of organic methylammonium cation into the lattice and layering a hydrophilic insulating polymer interface layer on top of the ZnO electron-injecting electrode, Wong created a high-quality CsPbBr_3_ perovskite thin film [23]. Extensive research has been conducted on the use of alkali and alkaline earth metals for the passivation and interface engineering of perovskite devices [21,24,25,26,27].

Most photoelectronic devices require a constant electrical power source for operation or performance enhancement. That is detrimental for applications of the sensor in embedded structures, where the device needs to be functional for long periods, and any additional device, such as a battery, can cause a detrimental intrusiveness effect in a composite structure. The self-powered architecture allows the sensor to work while embedded in a structure without needing batteries or power sources. Self-powered photovoltaic devices have recently attracted considerable attention [28,29,30,31,32,33,34,35,36,37]. However, the performance of self-powered devices still needs to be improved compared to traditional photovoltaic devices functioning under bias voltage [32,34]. The performance of self-powered PDs is heavily influenced by the quality of the intrinsic layer and the ability to separate and transport the photogenerated carriers, or the electron-hole pairs, excited by light illumination [10,13,38,39,40,41].

The integration of a self-powered, all-inorganic CsPbBr_3_ perovskite as a photo-absorbing layer and ZnS:Cu as a light-emitting ML layer is described in this paper. A simple vertical device structure of indium tin oxide (ITO)/tin (IV) oxide (SnO_2_)/magnesium iodide (MgI_2_(0–10 mg·mL^−1^)/CsPbBr_3_/carbon electrode was used to evaluate the device’s photo-detection performance. MgI_2_ was added to the SnO_2_/perovskite interface to modify it. The addition of MgI_2_ reduced interface defects and improved the performance of the self-powered ML-perovskite sensor. The device had a faster response time of 0.65 ms. The light emitted from the ML layer was efficiently collected and converted into distinct electrical signals for SHM applications, according to impact testing and mechanical three-point bending tests.

## 2. Materials and Methods

### 2.1. Materials

SnO_2_, 15% in H_2_O colloidal dispersion, and lead bromide (PbBr_2_) were purchased from Alfa Aesar. Anhydrous dimethyl sulfoxide (DMSO, 99.5%), and cesium bromide (CsI), were obtained from Sigma Aldrich. ZnS:Cu (GL29/B-C1) was purchased from Phosphor Technologies. All of the materials were used without further treatment.

### 2.2. Sensor Device Fabrication 

**Figure 1:**
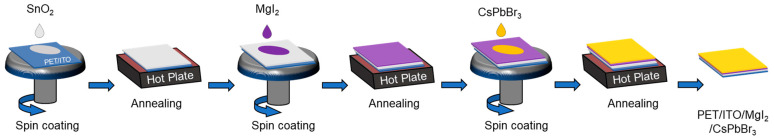
Fabrication procedure of the CsPbBr_3_ perovskite thin film.

Hydrochloric acid and zinc powder were used to etch ITO-coated polyethylene terephthalate (PET) substrates. The substrates were then cleaned with nano pure water, acetone, and isopropanol. After cleaning, the flexible substrates were treated for 5 min with oxygen plasma. SnO_2_ colloid precursor was diluted (1:6) with deionized water and stirred overnight. The SnO_2_ solution was spin-coated for 30 s at 3000 rpm onto PET/ITO substrates before being annealed in ambient at 100 °C for 60 min, followed by 5 min of plasma treatment. MgI_2_ was dissolved in methanol in various concentrations and spin-coated onto the PET/ITO/SnO_2_ substrates for 30 s at 3000 rpm before being annealed at 100 °C for 15 min. CsBr (0.3 M) and PbBr_2_ (0.3 M) were mixed in 1 mL DMSO to produce the CsPbBr_3_ precursor. The perovskite precursor was spin-coated on the substrates for 45 s at 1500 rpm before being annealed at 70 °C for 3 min and then at 105 °C for 20 min. Figure 1 depicts the one-step CsPbBr_3_ thin film fabrication procedure. Carbon paste electrodes were applied to the substrates with a doctor blade and dried for 15 min at 80 °C. The active area of the devices was set at 0.06 cm^2^.

The device was built using our previously described method for ML layer integration [4,6]. Using a Thinky mixer, the ZnS:Cu material was mixed into a polydimethylsiloxane (PDMS) elastomer. The ZnS:Cu/PDMS composite was deposited on the other side of the PET substrate using a doctor blade and heated until a total cure was obtained.

### 2.3. Multifunctional Composite Fabrication

**Figure 2:**
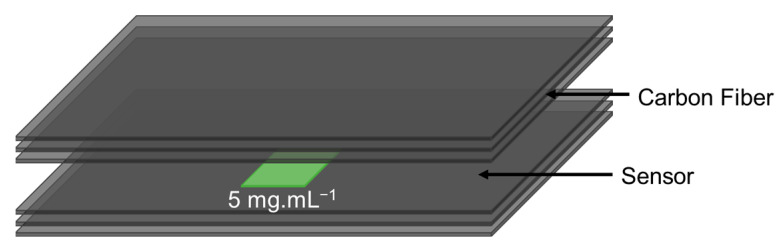
Schematic of the composite samples with embedded sensors for mechanical testing.

Multifunctional composites with embedded impact sensors were produced using plain weave carbon fiber fabric as reinforcing fibers and vinyl-ester resin as the system’s matrix. As shown in Figure 2, the sensors were embedded between the third and fourth carbon fiber plies. The data from the sensors were collected using embedded copper electrodes. Six layers of reinforcing fibers were used to create the final composite. Vacuum-assisted resin transfer molding (VARTM) infusion was used as usual.

### 2.4. Materials and Device Characterization

An Agilent Cary 5000 was used to obtain the ultraviolet-visible (UV-Vis) absorption spectra. The current-voltage (I–V) parameters were measured with a Keithley 2410 and LabView under a white light-emitting diode (LED) lamp with a 100 mW/cm^2^ intensity. The time-dependent response was obtained with a NI-6210 DAQ and boosted with a Hamamatsu C7319 on a low bandwidth setting and 10^5^ gain. The data were processed using MATLAB. The impact testing was carried out with the help of a customized drop-tower setup, and the cyclic 3-point bending test was carried out with the help of a Shimadzu mechanical testing system. The sensor’s response signal was collected using the same configuration for I–V measurements. 

## 3. Results

### 3.1. Optical Characterization

**Figure 3:**
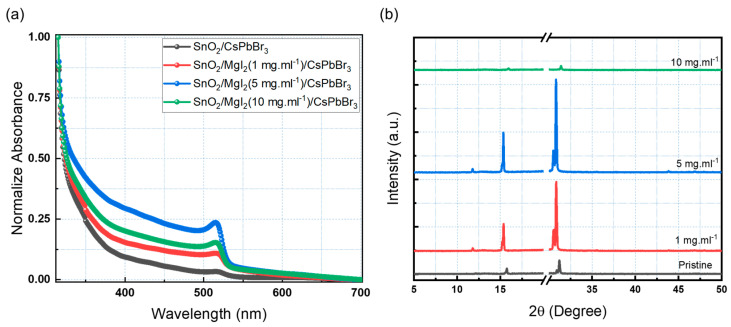
(**a**) Absorption spectra and (**b**) XRD spectra of the perovskite with different concentrations of MgI_2_.

MgI_2_ solution ratios of 1, 5, and 10 mg·mL^−1^ were used to investigate the ETL/perovskite interface modification. Mg^2+^ and I^−^ ions effectively inhibit the formation of deep trap states at the ETL/perovskite interface, promoting surface passivation and decreasing device carrier recombination. Mg^2+^ ions can also diffuse into the interstitial regions of the perovskite lattice, resulting in an active passivation action [21,24,27]. The UV-Vis absorption technique was used to investigate the optical properties of CsPbBr_3_ films with and without MgI_2_ treatment. As shown in Figure 3a, all samples had a sharp absorption edge of around 530 nm. Because of the absorption property of the CsPbBr_3_ films, they can be used as an active layer for visible photo-detection, particularly in the green light region. The MgI_2_ layer improved the absorption of the perovskite films, implying that the interface between the ETL and the perovskite was effectively modified. Further research was carried out to determine the effect of the interfacial modifier on the crystal quality of the inorganic CsPbBr_3_ perovskite. The crystallinity of the perovskite films was investigated using X-ray diffraction (XRD). Figure 3b depicts the XRD patterns of CsPbBr_3_ perovskite films on PET substrates. The XRD pattern’s break region attempts to remove the strong diffraction from the PET substrate. Because PET flexible substrates were used, the inorganic perovskite annealing temperature was kept constant at 105 °C. The intensities of the peaks increased as MgI_2_ concentration increased, indicating that the films were more crystalline. The XRD pattern of CsPbBr_3_ perovskite shows strong and prominent peaks with a high degree of crystallinity, which is beneficial for efficient charge transfer. Higher crystallinity, as well as larger grain size with fewer grain boundaries, are both indicated by a narrower and stronger X-ray diffraction peak, which is directly related to photovoltaic performance. Crystallinity was increased in thin films with 1 and 5 mg·mL^−1^ additions.

The crystallite size of the perovskite film was calculated using Scherrer’s equation, as shown in Equation (1) [42].

(1) $$D=\frac{K\lambda }{B\cos\theta }$$

where *D*, *K*, *λ*, *B*, and *θ* are the crystallite size (nm), Scherrer constant, X-ray wavelength (nm), Full Width at Half Maximum (FWHM) (radian), and XRD peak position (degree), in that order. Table 1 shows the calculated crystallite size for films with various MgI_2_ concentrations. The film containing 5 mg·mL^−1^ MgI_2_ had an average crystallite size of 74.54 nm, whereas the pristine films had 55.35 nm. These findings also suggested that the interface modification improved crystallinity and reduced defects, thereby improving the photophysical capabilities of the device.

**Figure 4:**
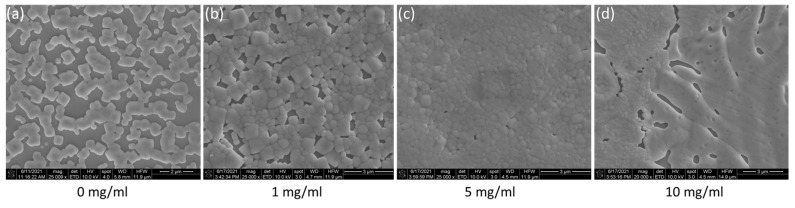
SEM images of perovskite films with (**a**) 0, (**b**) 1, (**c**) 5, and (**d**) 10 mg/mL of MgI_2_ interfacial layer.

SEM images of the obtained CsPbBr_3_ films with and without the addition of MgI_2_ at various concentrations were compared. The pristine CsPbBr_3_ film has some discontinuities and numerous pinholes, as shown in Figure 4. The film morphology significantly improves as the MgI_2_ concentration increases, with greater coverage uniformity and fewer minor pinholes. The thin film with a MgI_2_ concentration of 5 mg·mL^−1^ appears to have the best film coverage, with very compact grain boundaries and few pinholes. The films with a concentration of 10 mg·mL^−1^ showed signs of degradation, including poor film coverage and dissolved grain boundaries. The degradation of the films with a higher concentration of MgI_2_ may indicate saturation of the Mg layer, which prevents the perovskite layer from crystallizing properly. The disproportional addition of the compound can disbalance the perovskite crystallization, leading to an incomplete reaction and the formation of PbI_2_, preventing the full development of perovskite crystals on the film [23,43,44,45]. The SEM results agree with the UV-Vis and XRD characterization, indicating that adding MgI_2_ can help improve the morphology and crystallization of the CsPbBr_3_ film.

### 3.2. Electrical Characterization

**Figure 5:**
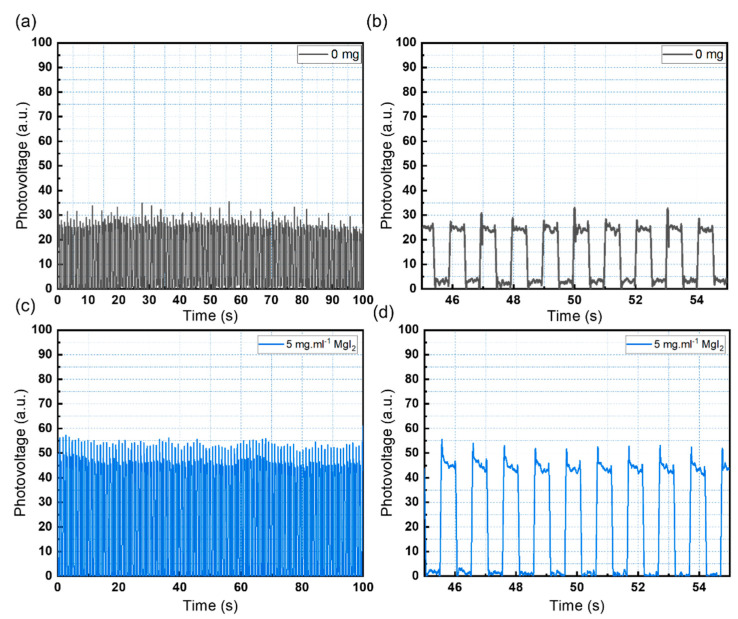
(**a**) On-off cycles of the pristine CsPbBr_3_ photodetector. (**b**) Enlarged detail of the pristine sensor response. (**c**) On-off cycles of the CsPbBr_3_ photodetector with 5 mg·mL^−1^ of MgI_2_. (**d**) Enlarged detail of the modified sensor response.

A vertical planar PET/ITO/SnO_2_/MgI_2_/CsPbBr_3_/carbon structure was used to investigate the photo-detection response of the CsPbBr_3_ photodiode. When light is absorbed by the perovskite layer, electron and hole pairs are separated. Electrons from the light-absorbing layer of the perovskite are carried into the conduction band of the perovskite, where they are injected into the SnO_2_ electron transport layer (ETL) and collected by the ITO electrode. To complete the electrical circuit, the carbon contact electrode collects the holes. Under a 0 V bias, the devices were tested with a pulsing white light LED at 1 Hz. Figure 5 depicts the devices’ consistent and stable response over time (a–d). The device’s response to the LED is consistent with the UV-vis and XRD data, which show that adding 5 mg·mL^−1^ of MgI_2_ improves sensor performance (Figure 5d). 

**Figure 6:**
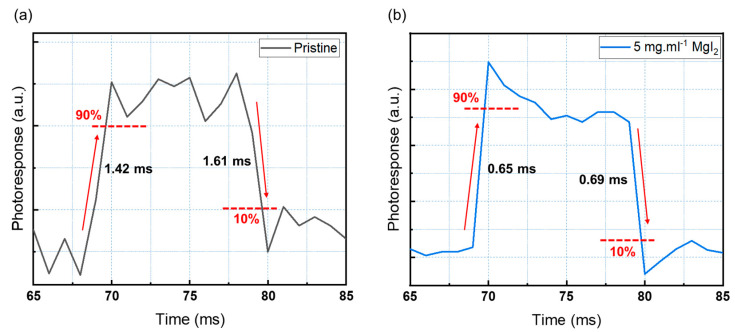
Rise and decay time of the (**a**) pristine device and (**b**) modified device.

The response speed of a photodiode is an important metric that reflects its ability to detect a rapidly changing optical signal [10,11,46,47]. High-performance photodiodes used in SHM must respond quickly and consistently to light illumination. The response time was evaluated using the previously described method. Under ambient conditions, a 470 nm pulse light source from an LED was controlled by a function generator with square waves at a frequency of 50 Hz to measure response time [11,46,47]. The reaction times of pristine CsPbBr_3_ photodiodes and devices incorporating 5 mg·mL^−1^ MgI_2_ are shown in Figure 6. The devices demonstrated a consistent and repeatable response with excellent performance. Figure 6 depicts the response rise (τ_rise_) and fall (τ_fall_) times, which are the times it takes for a photodiode to reach 90% and 10% of steady-state values, respectively (a, b). The device containing 5 mg·mL^−1^ MgI_2_ has a rise and fall time of 0.65 ms and 0.69 ms (Figure 6a), which is comparable to previously reported devices [47,48,49,50] but significantly faster than the pristine devices (Figure 6b). Table 2 summarizes the response time of different inorganic perovskite photodetectors.

### 3.3. Impact Sensing

A perovskite photodiode with a MgI_2_ layer (5 mg·mL^−1^) was combined with ZnS:Cu-PDMS and mechanically tested to evaluate the possibility of the modified CsPbBr_3_ devices for constructing ML-perovskite impact sensors. Light emission in ML materials is caused by mechanical stress. As a result, photon emission is predicted as the ML layer is stressed. The perovskite photodiode then captures the photons and converts them into an electrical current. Upon photoexcitation, electron-hole pairs are generated in the perovskite layer. Then, photogenerated carrier pairs, in the presence of the inherent electric field, are extracted and collected by the electron-transport layer and electrodes, generating electrical current [39,40,58]. The current fluctuation can be measured and correlated to the load applied to the device for sensing purposes. The change in the electrical current was expressed as follows:

(2) $$\frac{\Delta I}{I_{0}}=\frac{\left(I-I_{0}\right)}{I_{0}}$$

where *I* represents the electrical current measured during the test and *I*_0_ represents the sensor’s baseline electrical current, also known as the dark current. The mechanical energy applied to the composite is transmitted to the sensor, causing ML emission. The perovskite layer absorbs light and converts it into an electrical current. The intensity of an impact has a linear effect on the sensor signal [4,6].

**Figure 7:**
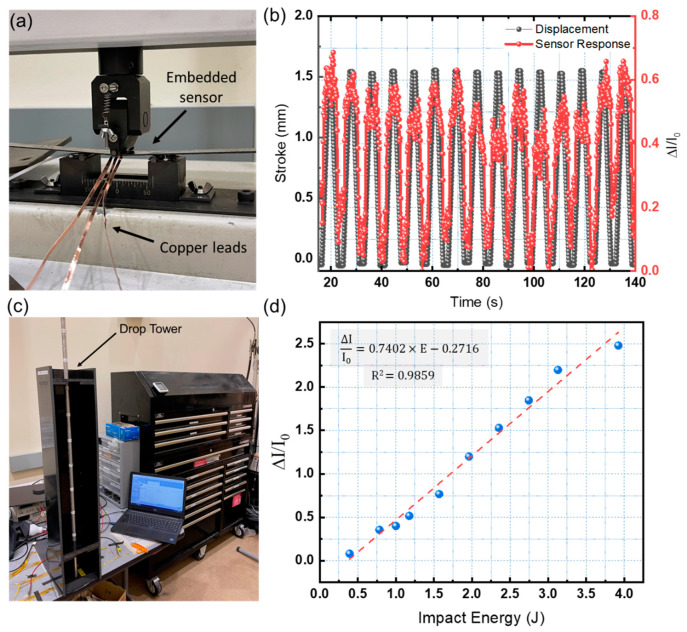
(**a**) Three-point bending testing setup. (**b**) Results of the three-point bending test. (**c**) Impact testing setup. (**d**) Sensor response for different impact energies.

A mechanical three-point bending test was performed to evaluate the capability of the modified ML-Perovskite sensor for SHM applications. Figure 7 depicts the three-point bending test configuration (a). The sensor was inserted into a carbon fiber composite sample and bent 250 times. Each bending cycle used a constant displacement of 1.5 mm. The results of a 15-cycle repeated bending test are shown in Figure 7b. The sensor generated distinct and visible signals for each cycle. Furthermore, the sensor intensity signals correlated well with the composite sample displacement. The sensor responded consistently across the cycles, indicating its potential for SHM applications.

To investigate the sensor’s potential for impact sensing, an in-house drop tower testing setup was used to impact a composite laminate with an embedded sensor with varying impact energies ranging from 0.4 J to 4 J. To evaluate the sensor’s low-energy impact-sensing capabilities, impact samples were subjected to ten successive impacts of increasing energies. The strikes were delivered directly to the surface of the composite structure, right on top of the sensor. The impact testing setup is depicted in Figure 7c. Drop-weight tower impact testing is a popular and widely used method for investigating low-velocity impact in composites and ML materials. The impact energy can be altered by adding mass to the impactor or adjusting the drop height. For each impact, all device configurations produced distinct signals. As shown in Figure 7d, the signal peak intensity is proportional to the impact energy. 

The minimum observable signal was obtained for an impact energy of 0.4 J. The minimal detectable impact energy could be reduced by improving the dark current of the perovskite photodetector. Table 3 summarizes some performance parameters of different ML-perovskite sensors.

A regression model was used to analyze the experimental data and is shown in the following equation:

(3) $$\frac{\Delta I}{I_{0}}=0.7402\times E-0.2716$$

where *E* is the impact energy value. The sensor’s sensitivity could be estimated as the change of sensor output due to the input parameter change (impact energy). The estimated sensitivity for the optimized sensor is 0.74 J^−1^**.** The load applied to the ML materials produces a linear relationship between pressure and light emission. 

The higher the energy applied to the ML crystals, the higher the expected emission of photons. The photocurrent of a perovskite photodetector increases linearly with increasing light intensity. As a result, the system’s output signal should rise linearly as the applied load increases. In other words, as the impact energy increases, the signal intensity increases, demonstrating that the sensor can be used for structural health monitoring of composite structures. The addition of MgI_2_ to the interface can increase the intensity of the resulting signal.

## 4. Conclusions

In conclusion, we discuss how an inorganic CsPbBr_3_ perovskite with a carbon-based electrode flexible photodetector can be utilized for the ML sensing and SHM of composite structures. The overall performance of the sensor was improved by the incorporation of MgI_2_ into the interfacial layer that is located between SnO_2_ and perovskite. The UV-vis data demonstrate that the device’s absorption spectrum has been enhanced, and as a result, it is now appropriate for monitoring ML emissions. The optimized flexible photodetector with a PET/ITO/SnO_2_/MgI_2_/CsPbBr_3_/carbon basic construction demonstrates excellent photoresponse when exposed to white light as the illumination source. According to the findings, the sensors could detect various loads throughout the composite structure. This enabled a correlation to be established between the sensor signal and the impact distance or composite displacement. The ML-perovskite sensor has demonstrated strong potential in SHM applications involving composite materials.

## Figures and Tables

**Table 1:** Crystallite size calculation for perovskite thin films with different concentrations of MgI_2_.

MgI_2_ Concentration (mg·mL^−1^)	Peak Position 2*θ* (^o^)	FWHM (^o^)	Average Crystallite Size (nm)
0 (pristine)	15.73	0.1434	55.35
1	15.37	0.1262	62.83
5	15.35	0.1064	74.54
10	15.90	0.1908	41.61

**Table 2:** Summary of the response time of different inorganic-based perovskite photodetectors.

Photodetector Structure	Fall/Rise Time	Ref.
ITO/SnO_2_/MgI_2_/CsPbBr_3_/Carbon	0.65 ms/0.69 ms	This work
ITO/SnO_2_/CsPbBr_3_/Carbon	1.42 ms/1.61 ms	This work
ITO/SnO_2_/CsPbBr_3_ MC/PTAA/Au	0.03 ms/0.039 ms	[51]
ITO/SnO_2_/CsPbBr_3_/Spiro-MeOTAD/Au	0.14 ms/0.12 ms	[52]
Au/CsPbBr_3_/Au	0.6 ms/0.9 ms	[53]
Au/CsPbI_3_/Au	24 ms/29 ms	[54]
ITO/CsPbBr_3_/ITO	0.5 ms/1.6 ms	[55]
Au/CsPbBr_3_/Au	0.2 ms/1.3 ms	[56]
ITO/PEDOT:PSS/Cs_3_Bi_2_I_6_Br_3_/C_60_/BCP/Ag	40.7 ms/27.1 ms	[57]

**Table 3:** Summary of the performance of different ML-perovskite sensors.

Sensor Configuration	Application	Min. Detected Load	Sensitivity	Ref
ZnS:Cu-PDMS/ITO-PET/SnO_2_/MgI_2_/CsPbBr_3_/Carbon	Impact/Three-Point Bending Sensing (Embedded Sensor)	0.4 J	0.74 J^−1^	This work
ZnS:Cu-PDMS/ITO-PET/SnO_2_/CsPbBr_3_/Carbon	Impact/Three-Point Bending Sensing (Embedded Sensor)	1 J	0.95 J^−1^	This work
ZnS:Cu-PDMS/ITO-PET/SnO_2_/MAPb(Br0.1I0.9)3/Carbon	Impact Sensing/Damage Localization (Embedded Sensor)	0.6 J	0.47 J^−1^	[7]
ZnS:Cu-PDMS/ITO-PET/SnO_2_/MAPb(Br0.1I0.9)3/Au	Impact/Pressure Sensing (Embedded Sensor)	100 kPa	0.137 kPa^−1^	[6]
ZnS:Cu-PDMS/ITO-PET/SnO_2_/MAPb(Br0.1I0.9)3/Spiro-oMeTAD/Au	Pressure Sensing (Surface-Attached Sensor)	11 kPa	0.095 kPa^−1^	[4]

## Data Availability

Data will be provided upon request.
